# A narrative review on ethical considerations and challenges in AI-driven cardiology

**DOI:** 10.1097/MS9.0000000000003349

**Published:** 2025-05-12

**Authors:** Dev Patel, Chandramouli Chetarajupalli, Saad Khan, Surayya Khan, Tirath Patel, Samuel Joshua, Richard M. Millis

**Affiliations:** a Lokmanya Tilak Municipal Medical College, Mumbai, India; b Zhengzhou University, Henan, PR China; c Saidu Medical College, Saidu Sharif, Pakistan; d Khyber Medical University, Peshawar, Pakistan; e School of Medicine, Trinity Medical Sciences University, Ratho Mill Kingstown, Saint Vincent and Grenadines; f Department of Clinical Anatomy and Medical Imaging, American University of Antigua, College of Medicine, Coolidge, Antigua; g Department of Medical Physiology, American University of Antigua College of Medicine, Antigua

**Keywords:** accuracy, artificial intelligence, cardiology, data privacy, ethics

## Abstract

**Introduction:**

Artificial intelligence (AI) is revolutionizing cardiology by enhancing diagnostic precision, prognostic accuracy, and treatment planning. Its integration raises ethical concerns like bias, privacy, accountability, and the risk of dehumanizing healthcare. This review focuses on navigating these challenges while maximizing AI’s potential in patient care.

**Methodology and aims:**

A narrative review was conducted to explore the ethical challenges associated with AI in cardiology. Key areas of focus included bias in training datasets, data privacy, the “black-box” nature of AI systems, and the need for transparency and accountability in clinical decision-making.

**Results and critical insights:**

AI improves accuracy in diagnosing and managing cardiovascular conditions but presents risks such as exacerbating healthcare disparities and challenges in patient data security. Strategies include creating ethical frameworks, integrating diverse datasets, and emphasizing the importance of clinician-AI collaboration to ensure equitable outcomes.

**Conclusion and limitations:**

AI offers transformative opportunities for cardiology, yet its success hinges on addressing ethical, technical, and regulatory challenges. Robust frameworks promoting fairness, transparency, and privacy are crucial. Limitations include a lack of real-world validation and the need for ongoing oversight to adapt to evolving clinical demands.

**HIGHLIGHTS**

- Integration of AI in cardiology: Evaluates how AI improves the precision of diagnosis and effectiveness of treatment in cardiology.
- Ethical challenges: Addresses important ethical issues such as prejudice in AI-driven cardiology, data privacy, and informed consent.
- Accountability and transparency: Stresses the necessity of explainable and transparent AI models in order to foster patient and healthcare provider confidence.
- Regulatory and economic barriers: Discusses the financial, legal, and technological obstacles to the use of AI in cardiology.
- Future directions: Makes recommendations for methods to guarantee the application of ethical AI and emphasizes the significance of ongoing research in this developing topic.

## Introduction

Artificial intelligence (AI) has recently attracted significant attention, leading to critical advancements in various fields including healthcare. In particular, large language models (LLMs), such as ChatGPT and Perplexity AI, are prominent examples of how AI reshapes the medical landscape. AI technologies offer transformative potential in patient data management, diagnostic accuracy, and personalized treatment plans, particularly in fields such as cardiology and radiology^[^1^]^. In cardiology, AI is revolutionizing the prediction and diagnosis of cardiovascular conditions, helping to identify intricate patterns that may be overlooked by human experts. Wearable and implantable devices powered by AI can collect extensive physiological datasets, which can assist in the diagnosis and management of cardiac diseases. In addition, machine learning algorithms are now being employed to analyze cardiac imaging, such as magnetic resonance imaging (MRI), computed tomography (CT) scans, and echocardiograms, with high precision. AI’s role of AI extends to interpreting electrocardiograms (ECGs) and assessing coronary artery blockages through fractional flow reserve CT, thereby minimizing unnecessary interventions^[^2^]^.

### Revolutionizing diagnosis and management

Cardiology is a medical field in which AI has made significant strides. AI-driven technologies can help clinicians predict cardiovascular events, diagnose conditions more accurately, and improve patient outcomes through personalized treatment plans. Wearable and implantable devices that utilize AI to continuously monitor patients are capable of collecting a vast amount of physiological data in real time. These data can be analyzed to identify potential cardiovascular risks early, allowing for timely intervention. AI also transforms the analysis of cardiac imaging, including echocardiography, MRI, and CT^[^3^]^. Machine learning algorithms can process these images with a precision level that often surpasses human capabilities, catching subtle abnormalities that may be missed in manual readings. Another significant advancement is the use of deep neural networks to analyze ECGs, helping diagnose various cardiovascular diseases more efficiently^[^4,5^]^. Fractional flow reserve computed tomography (FFR-CT) is an AI-based technology that assesses the degree of coronary artery blockage and reduces unnecessary invasive procedures. In cardiology, AI models like IBM Watson (Uses AI to analyze medical data, assisting in diagnosis, treatment planning, and personalized care) and TensorFlow (Machine learning framework used to develop predictive models for heart disease and analyze medical imaging) have been instrumental in uncovering patterns such as early indicators of cardiovascular events and disease progression through imaging and wearable device data. In another instance, EchoNet-Dynamic employs convolutional neural networks (CNNs) to analyze echocardiogram videos with precision rivaling expert cardiologists. Similarly, Ultromics’ EchoGo leverages AI for coronary artery disease prediction. These systems use advanced data processing techniques to assess functional impairments, offering diagnostic and prognostic insights that significantly enhance clinical decision-making. This ability to analyze large, complex datasets offers an unprecedented advantage in predictive and personalized healthcare management. The ability of AI to process and interpret large volumes of complex data has revolutionized the diagnosis and treatment of cardiovascular conditions. Despite these benefits, the widespread use of AI in cardiology raises several ethical concerns. These challenges are largely centered on bias, patient privacy, accountability for AI-generated decisions, and the potential dehumanization of healthcare^[^6,7^]^.

### Ethical concerns in AI-powered cardiology

AI systems, including those used in cardiology, are only as effective as the data on which they are trained. Inaccuracies or biases in the training data can lead to biased outcomes. For instance, if the data used to develop an AI model lack sufficient representation from minority groups or individuals with lower socioeconomic status, the model may generate less accurate predictions or diagnoses for these populations. In cardiology, this is particularly concerning because conditions such as hypertension, diabetes, and heart disease disproportionately affect marginalized populations. AI bias could exacerbate existing healthcare disparities by providing unequal care across different demographic groups. Addressing this issue requires the inclusion of diverse datasets that represent various ethnicities, sexes, and socioeconomic backgrounds to ensure that AI models perform equally across all patient populations^[^8,9^]^.

Second, the use of AI in cardiology often involves the processing of sensitive patient data, including continuous monitoring through wearable devices. The vast amount of data collected and processed by these systems raises serious concerns regarding patient privacy. As AI systems become more integrated with electronic health records (EHRs) and other health information technologies, the risk of data breach and unauthorized access to patient information increases. Ensuring that AI systems comply with stringent data protection regulations, such as the Health Insurance Portability and Accountability Act (HIPAA) in the United States, is essential. Additionally, patients must be informed about how their data are being used, and the consent processes should be transparent and comprehensive. The challenge lies in balancing the need for vast amounts of data to train AI systems with an ethical obligation to protect patient privacy^[^10^]^.

Third, as AI becomes more involved in clinical decision making, questions about accountability arise. Who is responsible for an AI system making an incorrect diagnosis or recommendation that leads to harm? The complex nature of AI systems, which often function as “black boxes,” renders it difficult to trace how specific decisions are made. This lack of transparency poses challenges in determining liabilities when errors occur. In the context of cardiology, where decisions can be life-saving or life-threatening, the stakes are particularly high. Although AI systems can provide valuable insights, they should not replace human judgment. Clinicians must retain the ultimate responsibility for decision making and ensure that AI recommendations are critically evaluated before implementation^[^11^]^.

Lastly, one of the most significant concerns regarding AI in healthcare is the potential for dehumanization. The introduction of AI technologies may lead to over-reliance on machine-generated data, reducing the emphasis on human aspects of care. In cardiology, a field that often requires nuanced clinical judgment and patient-centered care, over-reliance on AI could undermine the patient-clinician relationship. While powerful, can cannot replicate the empathy, intuition, and understanding that human clinicians bring to patient care. Maintaining a balance between AI-driven insights and human touch is critical to ensuring that the healthcare system remains patient-centered and compassionate^[^12,13^]^.

### Research aims

This review aimed to explore the ethical challenges posed by AI technologies in cardiology, particularly focusing on issues of bias, privacy, accountability, and regulation, and how these challenges affect clinical practice. Unlike general AI applications in healthcare, cardiology presents unique ethical dilemmas owing to its reliance on diverse datasets, real-time data collection from wearable devices, and the integration of AI into high-stakes decision-making processes. Furthermore, the review considers the need to balance AI advancements with human intelligence to avoid over-reliance on AI and the potential dehumanization of healthcare^[^14^]^. The integration of AI into cardiology holds great promise for improving the diagnosis, management, and treatment of cardiovascular diseases. However, these technological advancements have significant ethical challenges that must be addressed. To fully realize the potential of AI while mitigating these risks, it is essential to develop robust frameworks for ethical AI development and deployment. AI should complement, not replace, human expertise, preserving the essential human elements of care that are central to effective cardiology practice^[^15^]^.

- Explore bias in AI systems: Investigate how AI models in cardiology may exhibit bias against populations based on ethnicity, socioeconomic status, and other demographic factors and how this impacts healthcare outcomes.
- Assess patient privacy and data security: Examine the ethical challenges of protecting patient privacy and ensuring data security in AI-powered cardiology, particularly with the use of wearable and implantable devices.
- Exploring the accountability in AI decision-making: Assess the responsibility of healthcare providers and developers for AI-generated diagnoses and recommendations and explore frameworks for ensuring accountability.
- Examine transparency of AI technologies: Investigate the transparency of AI algorithms, focusing on the “black box” issue and the challenges it poses for understanding how decisions are made in cardiology.

Investigate the balance between AI and human care: Explore how to balance AI’s role of AI in cardiology with the human elements of care, ensuring that AI enhances rather than diminishes the patient-clinician relationship.

## Methodology

This narrative review has followed all the necessary guidelines.

A comprehensive literature search will be conducted across several electronic databases, including PubMed, the Cochrane Library, and Google Scholar, focusing on articles published between 2000 and 2024. The search strategy used a combination of terms and keywords related to AI, cardiovascular medicine, ethical considerations, regulations, and implementation challenges. Only peer-reviewed articles discussing the ethical challenges of AI in cardiology were included.

Table 1 shows all the selected studies to review in order to write this paper.

**Table 1:** Key findings

Name of the paper	Key findings
Lang M *et al*^[^38^]^	The study highlights critical legal and ethical challenges of AI in cardiovascular imaging, including issues with accountability, regulatory gaps, and intellectual property. Ethical concerns focus on AI’s “black-box” nature, which complicates transparency and trust, potential biases in datasets leading to disparities, and privacy concerns around patient data use. Addressing these challenges is essential to ensure equitable, safe, and responsible integration of AI into clinical practice.
Lewin S *et al*^[^39^]^	The study highlights ethical challenges and opportunities in using AI for cardiovascular medicine. Key concerns include bias in AI algorithms, data privacy, accountability, and disparities in access. It emphasizes the importance of transparency, regulatory frameworks, and interdisciplinary collaboration to ensure ethical AI integration. The authors advocate for patient-centric approaches, equitable AI deployment, and ongoing evaluation to maximize benefits while minimizing risks in cardiovascular healthcare.
Marey A *et al*^[^40^]^	The study highlights the transformative potential of generative artificial intelligence (AI) in cardiovascular imaging education. It emphasizes AI’s ability to personalize patient education, improve comprehension of complex imaging findings, and enhance decision-making through tailored visual and textual content. The authors advocate for integrating AI tools to bridge communication gaps between patients and clinicians, ultimately improving patient engagement, satisfaction, and outcomes in cardiovascular care.
Mooghali M *et al*^[^41^]^	This review revealed key ethical concerns and barriers and facilitators of trust in AI-enabled medical devices from patients’ and healthcare providers’ perspectives. Successful integration of AI into cardiovascular care necessitates implementation of mitigation strategies. These strategies should focus on enhanced regulatory oversight on the use of patient data and promoting transparency around the use of AI in patient care.
Ahmed M *et al*^[^42^]^	The article highlights the transformative potential of generative artificial intelligence (AI) in cardiovascular imaging, emphasizing its applications in image enhancement, anomaly detection, and personalized care. It also addresses key ethical and legal concerns, including data privacy, algorithmic bias, and transparency. The authors stress the importance of interdisciplinary collaboration and regulatory frameworks to ensure responsible implementation, enabling cardiovascular imagers to harness generative AI effectively while safeguarding patient safety and ethical standards.
Ferdowsi M *et al*^[^43^]^	The study emphasizes the development of a privacy-preserving and interpretable AI model for cardiovascular disease detection. Key findings include the successful integration of differential privacy to safeguard sensitive patient data and the application of explainable AI techniques to enhance clinical interpretability. The model demonstrates high accuracy while ensuring ethical and transparent decision-making, addressing critical challenges in adopting AI for healthcare applications.
Nolin-Lapalme A *et al*^[^44^]^	The study highlights the potential for bias in artificial intelligence (AI) models used in cardiology, stemming from disparities in data collection, processing, and interpretation. Key strategies for mitigating bias include ensuring diverse and representative datasets, implementing rigorous validation protocols, and increasing transparency in model development. The authors advocate for collaborative efforts among clinicians, data scientists, and policymakers to establish equitable and trustworthy AI applications in cardiac care.
Fenech ME *et al*^[^45^]^	This explores the ethical, social, and political challenges associated with AI in cardiac imaging in the UK. Key findings include concerns about data privacy, the potential for bias in AI algorithms, the need for transparency in decision-making, and the importance of maintaining clinician oversight. The authors emphasize the need for regulatory frameworks, professional education, and public engagement to ensure ethical AI integration in healthcare.

## Critical insights

The main aim of this narrative review was to provide insights into the use of AI in cardiology^[^16^]^. Many AI technologies are emerging in cardiology, including machine learning algorithms, deep neural networks, echocardiogram (echo) interpretation, and FFR-CT^[^17^]^. These technologies can assist providers in accurately interpreting cardiac images and making precise diagnoses. The development of tools such as deep neural networks can reduce human errors and oversights in the interpretation of ECGs, thereby enabling early diagnosis and prompt management^[^18^]^. Machine learning AI algorithms can decrease over-reliance on healthcare providers by facilitating the evaluation of CT, MRI, and echocardiography^[^19^]^. Implantable and wearable devices can revolutionize the management of patients with chronic heart conditions by collecting data over time and showing the progression or regression of the disease course^[^20^]^. Using these tools, we can predict the risk of cardiovascular diseases in patients and perform appropriate risk stratification. Advanced tools such as FFR-CT can reduce the burden of unnecessary cardiac interventions on the healthcare system by providing percutaneous interventions only to patients with significant functional blockage of the coronary arteries^[^21^]^. These technologies can improve the accuracy of interpretations, enhance the efficiency of physicians, and assist in providing personalized care to individual patients^[^22^]^.

### Ethical considerations in AI-driven cardiology

#### Informed consent

One of the key ethical challenges in the integration of AI into cardiology is obtaining informed consent from patients. Informed consent is a fundamental principle in healthcare that requires patients to be fully aware of and agree to the medical interventions they undergo. However, the technical complexity of AI presents unique difficulties in achieving this, particularly in explaining how AI algorithms function and influence medical decision-making. AI systems in cardiology often analyze vast amounts of patient data and generate predictions or recommendations that clinicians may rely on for diagnosis or treatment plans. Explaining these processes in layman terms is difficult because AI algorithms, such as machine learning models, are often seen as “black boxes,” and their decision-making processes are not easily understood, even by healthcare professionals. This makes it challenging for physicians to communicate with patients how AI reaches its conclusions, and what potential risks might arise from its use.

Patients have the right to understand how AI is involved in their care, but they may lack the technical background to grasp its full implications. This puts their autonomy at risk, as they might consent to AI-based interventions without fully understanding their consequences. For informed consent to be truly valid, health care providers must find effective ways to explain AI’s role of AI in patient care, including its benefits, limitations, and associated risks. To address this issue, health care professionals must develop clear and accessible communication strategies that make AI concepts comprehensible to patients. This could involve the use of visual aids, simplified language, or analogies that relate to everyday experiences. By doing so, patients can make informed decisions about their healthcare, ensuring that their autonomy and understanding are respected. Ultimately, enhancing the informed consent process in AI-driven cardiology will require collaborative efforts from clinicians, ethicists, and AI developers to promote transparency and patient-centered care^[^23^]^.

#### Data privacy and security

As AI technology has become increasingly integrated into cardiology, safeguarding patient data privacy and security has become a critical ethical consideration. AI-driven systems rely on vast amounts of sensitive medical data, often collected from wearable devices, electronic health records (EHRs), and other sources. These data are essential for training AI algorithms to make accurate diagnosis and treatment recommendations. However, the involvement of third-party AI systems increases the risk of data breaches, unauthorized access, and the misuse of sensitive patient information. Ensuring the security of patient data requires adherence to stringent data protection laws such as the Health Insurance Portability and Accountability Act (HIPAA) in the United States or the General Data Protection Regulation (GDPR) in Europe. These regulations mandate that healthcare providers and AI developers implement robust security measures including encryption, secure storage, and restricted access to medical information. As AI technologies continue to evolve, it is essential to continuously update these safeguarding tools to avoid new cybersecurity threats.

In addition to implementing technical safeguards, healthcare organizations must foster a culture of privacy to preserve patient trust. This involves educating healthcare providers and AI developers on the importance of ethical data-handling practices and ensuring transparency with patients about how their data are collected, processed, and used. Clear and informed consent processes should be in place to allow patients to make decisions regarding the use of their medical information in AI systems. By addressing these concerns, the healthcare industry can mitigate the risks associated with AI-driven cardiology, while ensuring the ethical use of patient data. Ultimately, maintaining high standards of data privacy and security will help protect patient autonomy, build trust in AI technologies, and support the integration of AI into clinical practice^[^24^]^.

#### Bias and fairness

The integration of AI into cardiology presents significant ethical concerns, particularly regarding biases and fairness. AI systems used in clinical decision making are often trained on large datasets. However, if these datasets lack diversity or contain inherent biases, the resulting AI models may exhibit biased outcomes, leading to unequal treatments and disparities in patient care. For example, if the data used to train an AI model predominantly represent specific demographics such as those from higher socioeconomic backgrounds or certain ethnicities, the AI system may perform poorly when diagnosing or treating underrepresented populations. This could further exacerbate existing healthcare disparities, particularly in marginalized groups. To address these ethical challenges, AI algorithms must be rigorously tested and validated across diverse demographic groups. Ensuring that the datasets are representative of various ethnicities, genders, and socioeconomic statuses is crucial for creating more equitable AI models. Additionally, the continuous monitoring of AI systems in clinical practice is essential to detect and mitigate biases that may emerge over time. This process should include assessing the impact of AI-driven decisions on different patient populations and refining algorithms as needed.

Incorporating fairness into AI systems also requires the development of next-generation algorithms with built-in bias-detection and correction mechanisms. These systems would automatically flag and adjust for bias to ensure more equitable outcomes. Moreover, healthcare professionals should collaborate with AI developers to ensure that fairness and inclusivity are prioritized during the algorithm-development phase. This collaboration could also involve creating regulatory frameworks that mandate fairness audits and accountability for AI models before they are deployed in a clinical setting. Ultimately, addressing bias and fairness in AI-driven cardiology is crucial to ensure that these technologies do not perpetuate healthcare inequalities. By promoting diversity in training data and implementing robust bias-mitigation strategies, AI can be harnessed to improve cardiovascular care for all patients regardless of their background^[^24^]^.

#### Transparency and explainability

The growing use of AI in cardiology has introduced significant ethical concerns, particularly regarding transparency and explainability. AI systems, particularly complex models such as deep learning, are often regarded as “black boxes” because their decision-making processes are difficult to interpret. In a field as critical as cardiology, where clinical decisions can be life-saving, the inability to understand how an AI system reaches a particular conclusion raises concerns regarding trust and accountability. Transparency is essential for the ethical integration of AI into cardiology. Physicians and patients alike must comprehend the rationale behind AI-generated diagnoses or treatment recommendations. Without this clarity, it is challenging for healthcare providers to fully trust AI systems or make informed clinical decisions based on their outputs. Moreover, the lack of transparency may lead to over-reliance on AI, reducing the clinician’s involvement in critical decision making and potentially undermining patient care.

Efforts must be made to develop interpretable and explainable models to foster trust in AI technologies. These systems should not only deliver results but also provide understandable explanations that clinicians can review and validate. Techniques such as interpretable machine learning (IML) and post hoc analysis are being explored to make AI outputs more transparent, offering insights into how the model arrived at its conclusions. Such transparency ensures that AI-driven decisions are clinically sound and ethically justified, allowing healthcare providers to make informed judgments. In cardiology, where the conditions and treatments are often complex, explainable AI is crucial for patient safety and ethical practice. By increasing transparency and ensuring that AI systems are interpretable, healthcare providers can maintain a more collaborative and trusting relationship with both patients and AI technologies, ultimately enhancing the quality of care delivered in a field that is central to life-saving interventions^[^24^]^.

#### Accountability and liability

The integration of AI into cardiology presents profound ethical considerations, particularly regarding accountability and liability for decisions made using AI models. As AI systems become increasingly involved in diagnosing and managing cardiovascular conditions, the question of responsibility for errors or adverse outcomes has become critical. The complexity of AI algorithms, which often operate as “black boxes,” complicates the attribution of accountability. In instances where AI systems provide erroneous recommendations or diagnoses, determining who is responsible—healthcare providers, AI developers, or institutions—poses significant legal and ethical challenges. The current legal frameworks may not adequately address these nuances. As AI technologies evolve, they require the development of new legal standards and regulations to clearly define the roles and responsibilities of all parties involved. This adaptation is essential to ensure that accountability is appropriately shared among healthcare providers who implement AI recommendations, developers who create the algorithms, and institutions that deploy these technologies in clinical settings.

To overcome these challenges, it is imperative to establish comprehensive guidelines for the ethical use of AI in cardiology. This includes clear documentation procedures that outline how AI-generated decisions are to be recorded and evaluated as well as decision-making protocols that integrate AI insights while preserving human oversight. By delineating responsibilities, these guidelines can help mitigate the risk of liability disputes and ensure that patient safety is paramount. Furthermore, fostering a culture of transparency and collaboration between stakeholders can enhance accountability in AI-driven cardiology. Continuous training and education for healthcare providers regarding the capabilities and limitations of AI technologies can empower them to make informed decisions and advocate for patient well-being. Ultimately, as AI continues to reshape the landscape of cardiology, addressing accountability and liability is essential for maintaining ethical standards and safeguarding the interests of patients in this rapidly evolving field^[^24^]^.

### Challenges in implementing AI in cardiology

#### Technical challenges

The integration of AI into cardiology presents several technical challenges that must be addressed to realize its full potential. One of the primary hurdles is ensuring that AI systems are compatible with diverse medical workflows and existing digital-record systems. In cardiology, clinicians rely on various technologies and platforms for patient management, and the seamless integration of AI tools is crucial for their effective utilization. Without interoperability, AI adoption can be hindered, leading to inefficiencies and potential disruptions in clinical practice. Another significant technical challenge is the need for rigorous validation of the AI algorithms. For AI systems to be deemed accurate and reliable, they must undergo comprehensive testing in a real-world clinical setting. This involves not only assessing the initial performance of AI models, but also establishing protocols for continuous performance monitoring. Regular evaluations are essential to ensure that AI tools maintain their effectiveness over time, especially as new data emerge and clinical practice evolves.

Data quality and completeness are critical factors influencing the success of AI implementation in cardiology. AI models require large datasets for effective training, and any gaps or inconsistencies in the data can significantly impact the performance of the algorithms. Incomplete or poorly curated data can lead to inaccurate predictions and suboptimal clinical recommendations, undermining the trust that healthcare professionals place on AI technologies. Ensuring high-quality data collection, standardization, and management is vital for maximizing the potential of AI in diagnosing and managing cardiovascular diseases. Moreover, the dynamic nature of cardiovascular care, characterized by rapidly changing guidelines and treatment protocols, poses an additional challenge for AI integration. AI systems must be adaptable and responsive to evolving clinical knowledge to be relevant and beneficial. Overcoming these technical challenges requires collaborative effort among clinicians, data scientists, and technology developers to create robust, reliable, and user-friendly AI solutions tailored to the needs of cardiology^[^25^]^.

#### Regulatory barriers

The implementation of AI in cardiology holds immense potential; however, it also faces significant regulatory challenges. The current regulations governing AI in healthcare are still in their early stages, and existing guidelines do not sufficiently cover the unique aspects and complexities of AI technologies. As AI continues to evolve, it is crucial to develop updated and comprehensive regulatory frameworks to ensure safe and effective use in cardiology. One of the primary regulatory challenges is ensuring patient safety while incorporating AI into clinical practice. AI systems in cardiology often handle large amounts of sensitive patient data such as real-time physiological monitoring from wearable devices or imaging data from echocardiograms and CT scans. Protecting these data while maintaining their utility for AI-driven insights is essential. However, currently, there are no universally accepted standards to guarantee that AI systems handle patient data responsibly and securely.

Another critical challenge is the development of clear protocols for AI approval and implementation in cardiology. Unlike traditional medical devices and pharmaceuticals, AI systems are dynamic and may require continuous updates and recalibration based on new data. This presents a unique challenge for regulatory bodies, who must create flexible yet stringent protocols that allow AI to evolve without compromising patient safety. Determining how to regulate AI’s decision-making processes, particularly in life or death situations common in cardiology, adds another layer of complexity. Furthermore, AI in cardiology faces the challenge of balancing innovation and patient protection. Regulators must ensure that AI technologies do not introduce new risks or exacerbate the existing healthcare disparities. For example, AI systems trained on biased datasets can provide unequal care to different populations. Regulatory frameworks must address these risks by enforcing transparency in AI development and ensuring that models are tested across diverse patient groups before being widely implemented.

In conclusion, implementing AI in cardiology requires updated, well-defined regulatory protocols that prioritize patient safety, data privacy, and equitable care, while allowing for innovation and continuous improvement of AI systems^[^25^]^.

#### Clinical adoption

Despite the potential of AI to revolutionize cardiology, its clinical adoption faces several challenges. One of the primary barriers is skepticism among healthcare professionals regarding AI’s reliability and fear of job displacement. Many clinicians are concerned that AI may undermine their expertise or make human judgments less relevant. This apprehension stems from a lack of understanding of AI’s capabilities and limitations, as well as concerns about the “black box” nature of some AI algorithms, which makes it difficult to trace how specific clinical decisions are made. Another challenge is the perceived risk of over-reliance on AI systems. Cardiology often involves complex decision making that requires a nuanced understanding of patient history, preferences, and contextual factors that AI may not fully grasp. Clinicians may fear that AI could lead to dehumanized care, where data-driven decisions outweigh personalized patient care. This fear can make practitioners hesitant to integrate AI technologies into their practice, even when AI can improve the diagnostic accuracy or treatment planning.

To address these concerns, comprehensive education and training of healthcare professionals is essential. Emphasizing AI’s role of AI as a tool to enhance rather than replace clinical expertise is crucial for fostering acceptance. By demonstrating how AI can improve efficiency, reduce cognitive burden, and enhance diagnostic precision, clinicians can better appreciate the complementary role of AI in patient care. Real-world evidence demonstrating AI’s success of AI in cardiology, such as its ability to accurately predict cardiovascular events or analyze complex imaging data, can further support its adoption. Highlighting successful use cases will help to shift the perception of AI from a potential threat to a valuable tool that enhances clinical outcomes.

In summary, addressing concerns about AI’s reliability, providing proper education, and demonstrating its real-world benefits are critical steps toward overcoming the challenges of its clinical adoption in cardiology^[^25^]^.

#### Economic obstacles

The integration of AI in cardiology holds the potential to revolutionize diagnosis, treatment, and patient care. However, economic challenges present significant barriers, particularly for small institutions. The cost of implementing AI technologies is a major concern, as it often requires substantial initial investments in infrastructure, software, and hardware. These costs include purchasing advanced AI platforms, upgrading systems to handle large datasets, and integrating AI tools with existing healthcare systems, all of which can be prohibitively expensive for institutions with limited budget. Beyond the initial investment, the continuous maintenance of AI systems adds a financial burden. AI tools must be regularly updated and maintained to ensure accuracy and reliability, which requires ongoing investment in both the software and hardware. Furthermore, specialized staff is essential for managing and optimizing AI technologies. Healthcare institutions must hire or train data scientists, AI specialists, and IT professionals to maintain these systems in addition to operational costs.

While AI promises to improve efficiency and reduce long-term healthcare costs, its implementation may initially increase expenses owing to the required technological investments. Smaller hospitals and clinics, especially in underserved areas, may struggle to justify the cost of AI integration without substantial financial support or clear evidence of a return on investment. Additionally, the economic implications of AI extend beyond the institutions. There are concerns regarding how AI affects the allocation of healthcare resources. While AI has the potential to streamline operations, reduce diagnostic errors, and improve patient outcomes, there is a risk that upfront costs could increase healthcare disparities, as wealthier institutions may adopt these technologies more readily than their less affluent counterparts. Addressing these economic challenges requires the development of cost-effective solutions and funding models, such as government subsidies, partnerships, and scalable AI systems, to ensure equitable access to AI technologies in cardiology across all healthcare settings^[^25^]^.

### Case studies and real-world examples

#### Successful implementations

AI has revolutionized various aspects of healthcare, with cardiology being one of the fields in which its application has seen tremendous success. AI technologies are driving significant advancements in cardiac care, from improving the diagnostic accuracy to optimizing treatment plans. Numerous case studies and real-world examples highlight how AI has been successfully integrated into clinical practice, offering insights into the potential of these technologies to enhance patient outcomes, streamline workflows, and reduce healthcare costs. This article explores several specific instances of AI’s successful implementation of AI in cardiology.

a. AI-Powered Diagnostic Tools for Early Detection

One of the most important uses of AI in cardiology is its application in early disease detection. AI algorithms have demonstrated remarkable accuracy in identifying cardiovascular diseases at an early stage, improving prognosis, and reducing the burden on healthcare systems. A notable example is the use of deep learning algorithms to analyze ECGs. A 2019 study used an AI model trained on over half a million ECGs to detect asymptomatic left ventricular dysfunction (ALVD), a condition that can lead to heart failure if left untreated. The AI was able to detect early signs of ALVD in patients who would otherwise have been considered healthy, achieving an accuracy comparable to that of traditional imaging techniques such as echocardiography. Early detection allows for timely intervention, which can significantly improve patient outcomes.

Another case study demonstrated the power of AI to detect arrhythmias using wearable devices. Team developed an AI algorithm capable of analyzing data from the smart watch’s heart rate sensor. The algorithm was able to detect atrial fibrillation, a common and potentially life-threatening arrhythmia, with impressive accuracy. This innovation not only improved early detection rates, but also provided a non-invasive, accessible solution for continuous heart monitoring, enabling earlier diagnosis and treatment for thousands of patients.

b. AI in Echocardiography: Enhancing Precision and Efficiency

Echocardiography, a key diagnostic tool in cardiology, has benefited significantly from AI integration. Traditional echocardiography relies heavily on the skill and experience of the clinician, which can lead to variability in results. However, AI algorithms can standardize the analysis process, improve accuracy, and reduce inter-observer variability.

For example, the EchoNet-Dynamic AI system uses deep learning to analyze echocardiograms with unprecedented precision. This AI tool was trained on thousands of echocardiogram videos to measure heart function, particularly left ventricular ejection fraction (LVEF), a critical marker of cardiac health. EchoNet-Dynamic achieved a level of accuracy comparable to that of expert cardiologists while streamlining workflows by reducing the time needed to analyze and interpret scans. By automating routine tasks, this technology allows clinicians to focus on more complex cases and improve overall efficiency in cardiac imaging departments.

Similarly, Ultromics, a UK-based company, developed AI algorithms specifically for echocardiographic analysis to predict coronary artery disease. Their AI system, known as EchoGo, uses deep-learning models to assess heart function and predict patient outcomes. A clinical study published in 2020 demonstrated that EchoGo could outperform human clinicians in predicting heart disease, achieving higher sensitivity and specificity. The ability of AI systems, such as EchoGo, to detect subtle abnormalities in heart function has far-reaching implications for improving diagnostic accuracy and patient care.

c. AI in Coronary Artery Disease Assessment

FFR-CT is another field in which AI has significantly impacted cardiology. FFR-CT is used to non-invasively assess the severity of coronary artery blockages by estimating the blood flow in coronary vessels. HeartFlow, a leading company in this space, developed an AI-powered FFR-CT analysis tool that helps cardiologists determine whether patients require invasive interventions, such as stenting or coronary artery bypass surgery.

The HeartFlow AI platform analyzes CT scans of the coronary arteries and generates a 3D model of the patient’s heart, allowing clinicians to visualize blockages and assess their functional significance. Studies have shown that HeartFlow’s AI technology can accurately predict which patients require invasive treatment, thereby reducing unnecessary procedures and improving patient outcomes. In a large-scale clinical trial known as the PLATFORM study, the use of HeartFlow reduced the need for invasive coronary angiography by 61%, demonstrating its effectiveness in guiding clinical decision making.

d. Real-Time Monitoring with AI-Enabled Wearables

Wearable devices have become increasingly common in cardiology, providing real-time monitoring of patient heart health. AI algorithms have enhanced the capabilities of these devices, allowing them to detect abnormal patterns and alert patients and clinicians in real-time.

The Apple Heart Study is a prime example of how AI-enabled wearables can transform cardiac care. In this study, over 400,000 participants used the smart watch to monitor their heart rate. The AI algorithm analyzed the heart rate data and alerted participants if an irregular rhythm was detected, prompting them to seek medical evaluation. This study demonstrated that the AI-powered wearable device could effectively identify atrial fibrillation, leading to earlier diagnosis and treatment. The use of wearables equipped with AI not only empowers patients to take control of their health but also provides clinicians with continuous data to improve diagnosis and management.

The successful implementation of AI in cardiology is evident from multiple case studies and real-world examples. AI has proven to be a game-changer in cardiac care by improving the early detection of cardiovascular diseases to enhance the precision of diagnostic tools such as echocardiograms and FFR-CT. These technologies not only offer enhanced diagnostic accuracy but also improve workflow efficiency, reduce healthcare costs, and provide real-time monitoring. As AI continues to evolve, its integration into cardiology holds great promise for further advancing patient outcomes and revolutionizing the diagnosis and management of cardiovascular diseases. The future of AI in cardiology is bright, and continued research and development will be key to unlocking its full potential^[^23^]^.

#### Ethical dilemmas

The integration of AI in cardiology has demonstrated significant potential for enhancing patient care; however, it has also revealed critical ethical dilemmas that must be addressed. Real-world scenarios highlight these ethical challenges, particularly those concerning bias, data representation, and accountability.

One notable case is the implementation of an AI system designed to predict cardiovascular disease risk based on electronic health records (EHRs). In one instance, a study found that the algorithm inadvertently favored certain demographic groups, particularly white patients, while underrepresenting minority groups. This bias was attributed to the training data, which were not sufficiently diverse, leading to unequal risk assessment. As a result, healthcare providers using this AI tool may have misdiagnosed or overlooked high-risk patients from underrepresented groups, potentially exacerbating the existing health disparities. To rectify this, the algorithm was retrained with a more representative dataset incorporating data from a wider demographic spectrum. In addition, ongoing monitoring was established to assess the performance of the system across different populations, demonstrating the necessity of continuous oversight in AI applications.

Another ethical dilemma emerged in the context of wearable devices equipped with AI algorithms for the real-time monitoring of cardiac patients. One case reported that patients with a lower socioeconomic status experienced a lack of access to the required technology, which created an uneven playing field for receiving AI-enhanced care. These disparities have raised concerns about equitable access to AI-driven health innovations. In response, healthcare systems have begun exploring ways to provide affordable access to such technologies, including community outreach programs and partnerships with local organizations to distribute wearables to underserved populations. This case highlights the ethical imperative to ensure that advancements in AI benefit all patients regardless of their socioeconomic background.

The introduction of AI systems raises questions regarding accountability. In one incident, an AI tool used for interpreting echocardiograms misidentified a critical condition, leading to delayed diagnosis and adverse patient outcomes. The ensuing debate centered on whether the responsibility for the error lay with AI developers, healthcare providers who relied on the tool, or the institutions that implemented it. This case emphasizes the importance of establishing clear guidelines for accountability and ensuring that healthcare providers remain vigilant and critically evaluate the AI-generated recommendations. These case studies exemplify the ethical dilemmas inherent in using AI in cardiology, underscoring the urgent need for proactive measures to ensure the responsible and equitable use of these technologies. By prioritizing diversity in training data, addressing access disparities, and clarifying accountability, stakeholders can work toward harnessing AI’s full potential while mitigating its ethical risks^[^24^]^.

### Strategies for addressing ethical challenges and recommendations for policy makers

The integration of AI in cardiology has opened significant avenues for enhancing patient care, improving diagnostic accuracy, and optimizing treatment strategies. However, this technological advancement comes with its own ethical challenges. As AI continues to evolve and integrate into healthcare systems, it is vital to establish robust strategies to address these ethical concerns. This article outlines key strategies for addressing the ethical challenges associated with the use of AI in cardiology, focusing on the development of ethical frameworks, ensuring transparency, promoting equitable healthcare, and strengthening data governance. Creating a comprehensive ethical framework for the integration of AI into cardiology is crucial to guide its implementation and usage. This framework should encompass several key factors that address ethical considerations. These factors include fairness, accountability, transparency, privacy, and safety^[^26^]^.

#### Fairness

Fairness is a foundational principle that emphasizes the need for equitable treatment across diverse patient populations. AI systems must be designed to minimize biases that could lead to disparities in healthcare delivery. In cardiology, this ensures that algorithms are trained on diverse datasets that accurately represent the population served. This includes considering variables such as age, sex, ethnicity, and socioeconomic status to avoid perpetuating existing inequalities.

#### Accountability

Accountability is essential for AI applications in cardiology. Clear lines of responsibility must be established for the decisions made with the assistance of AI systems. This includes defining the roles of health care providers, AI developers, and regulatory bodies. Mechanisms for reporting and addressing errors or adverse outcomes must be integrated into clinical practice to ensure accountability is maintained.

#### Transparency

Transparency in AI algorithms is vital for fostering trust between healthcare professionals and patients. This involves not only making the algorithms understandable but also providing clear explanations for how decisions are made. By developing a comprehensive ethical framework, stakeholders can collectively address the ethical implications of AI in cardiology, ultimately enhancing patient care and promoting responsible innovations.

An effective method to promote transparency is the use of open-source algorithms. Open-source algorithms promote collaboration and shared knowledge in AI development by allowing users to inspect and understand underlying methodologies and codes. This transparency can facilitate a better understanding and allow researchers and practitioners to identify potential biases or limitations in algorithms^[^27^]^.

#### Comprehensive documentation

A comprehensive documentation of AI methods is essential for understanding the various components of AI systems. This includes detailed data processing methods, data sources, preprocessing techniques, and rationales behind decision-making processes. By providing thorough documentation, users can better comprehend how AI systems function and the reasoning behind their outcomes. Such transparency not only builds trust among healthcare providers, but also empowers them to make informed decisions regarding patient care.

#### Continuous updates of AI models

The medical landscape is continuously evolving, and AI models must be updated to remain relevant and accurate. Regular updating of these models ensures that they reflect the latest medical knowledge, guidelines, and patient data. Continuous improvements also allow for the identification and rectification of biases that may arise over time.

#### User-friendly interfaces

Developing user-friendly interfaces can significantly enhance our understanding of AI-generated outcomes. Interfaces should be designed to present information in a clear and accessible manner, allowing clinicians to easily interpret results and integrate them into their decision-making processes. User-friendly interfaces foster a collaborative relationship between AI systems and healthcare providers, ultimately improving patient care^[^28^]^.

#### Ensuring equitable healthcare

Addressing bias and ensuring equitable healthcare delivery are essential components for integrating AI into cardiology. Various strategies can be implemented to promote fairness and inclusivity of AI applications.

#### Collecting diverse and representative data

One of the most effective strategies for mitigating bias in AI systems is to collect diverse and representative data from various populations. This means actively seeking data from underrepresented groups to ensure that the algorithms are trained on a wide range of patient demographics. By doing so, AI systems can produce more accurate and equitable outcomes, thereby reducing the risk of exacerbating healthcare disparities.

#### Assembling diverse groups for audits

Creating audits that involve diverse groups from different communities can reveal biases that may not be apparent in homogeneous datasets. These audits can help identify potential shortcomings of AI algorithms and provide valuable insights for improvement. Engaging with community representatives and stakeholders ensures that the perspectives of diverse populations are considered during the development and evaluation of AI systems.

#### Implementing policies for transparency and collaboration

Developing policies that promote transparency and collaboration is vital for ensuring equitable healthcare delivery. Such policies should encourage collaboration among AI developers, healthcare providers, and patients to create AI systems that prioritize fairness and inclusivity. Regular audits and evaluations of AI systems can help identify and address biases proactively, thereby contributing to more equitable healthcare outcomes.

#### Strengthening data governance

Strengthening data governance is a critical strategy for addressing ethical challenges associated with AI in cardiology. This involves the development of robust policies and practices related to data privacy, security, and consent.

Organizations must establish comprehensive data governance policies that outline privacy standards, access controls, and compliance requirements. These policies should be transparent and accessible to all stakeholders to ensure that data are well protected and handled responsibly. Stakeholders must understand their role in data governance to maintain patient trust and ensure compliance with ethical standards^[^29^]^.

#### Incorporating privacy measures

Incorporating privacy measures into the design phase of data management processes is essential to safeguard patient information. By adopting a privacy-by-design approach, organizations can ensure that data privacy is a fundamental consideration in every aspect of AI development and implementation. This approach helps mitigate the risks associated with data breaches and unauthorized access.

#### Regular training and awareness programs

Ongoing training and awareness programs for health care providers, data managers, and AI developers are crucial for promoting ethical data governance. These programs should focus on best practices for data handling, privacy standards, and ethical implications of AI in cardiology. By fostering a culture of ethical awareness, organizations can strengthen their commitment to responsible AI usage^[^30^]^.

#### Data privacy and security

One of the primary concerns in AI-powered cardiology is safeguarding patient data. The vast amounts of sensitive health information collected through wearable devices, imaging devices, and electronic health records (EHRs) must be stored and processed securely. Implementing strict data protection regulations, such as the Health Insurance Portability and Accountability Act (HIPAA) in the United States, ensures that patient data are handled with utmost care. AI developers and healthcare institutions must ensure encrypted data storage, restricted access, and secure data source. In addition, clear patient consent protocols are required to inform patients about how their data will be used, ensuring transparency and trust. By adopting rigorous cybersecurity measures, institutions can prevent data breach and maintain the confidentiality of sensitive health information. While AI can assist in decision making, human clinicians must retain the ultimate responsibility for patient care. AI recommendations should be critically evaluated by healthcare providers before being acted upon. Establishing clear guidelines for the use of AI and defining accountability in cases of AI-related errors are crucial for maintaining ethical standards in cardiology. These strategies will help ensure the responsible integration of AI in cardiology, protecting patient rights while leveraging AI’s benefits of AI for cardiovascular care^[^31^]^.

The integration of AI into cardiology presents significant opportunities for enhancing patient care, but also poses complex ethical challenges. Addressing these challenges requires a multifaceted approach, which includes developing comprehensive ethical frameworks, ensuring transparency, promoting equitable healthcare, and strengthening data governance. By implementing these strategies, stakeholders can foster trust, minimize biases, and ensure that AI technologies contribute to the advancement of patient-centered care in cardiology. Ultimately, ethical considerations must remain at the forefront of AI development and implementation, ensuring that technological advancements align with the values and needs of patients and healthcare providers alike^[^32^]^.

### Future directions and recommendations

AI has revolutionized various fields, including cardiology, by enhancing diagnostic precision, treatment efficacy, and overall patient outcomes. The ability of AI to process vast datasets, such as those from electronic health records (EHRs) and advanced imaging techniques, is transforming the diagnosis and management of cardiovascular conditions. With its growing role in cardiology, AI is now capable of identifying patients at risk of conditions such as coronary artery disease and heart failure, allowing for timely interventions. Moreover, AI-powered wearable devices enable continuous heart monitoring, providing real-time data that support preventive care. Despite these promising developments, significant areas for growth and investigation remain to ensure the ethical and effective integration of AI in cardiology. This section outlines the future directions and research priorities necessary to optimize the use of AI in cardiovascular care^[^33^]^.

#### Advancing predictive analytics in cardiovascular health

One of the most promising applications of AI in cardiology is its ability to predict cardiovascular events prior to their occurrence. By analyzing large datasets from EHRs, AI models can identify patients at risk of developing conditions such as coronary artery disease, heart failure, and arrhythmias. Future research should focus on refining the predictive models to improve their accuracy and reliability. This involves training AI systems with diverse datasets that represent various demographic groups to ensure that predictions are not biased or skewed toward specific populations. Additionally, more studies are needed to explore how AI can incorporate lifestyle factors such as diet, physical activity, and smoking into predictive models to create a more comprehensive risk profile for cardiovascular diseases.

AI’s role of AI in predictive analytics can be expanded by integrating genetic data and biomarkers into its algorithms. By leveraging genetic information, AI can help identify individuals with hereditary predispositions to heart disease, thereby allowing for earlier and more personalized interventions. Research on how genetic data can be securely integrated with AI models without compromising patient privacy is an important area for future studies^[^34^]^.

#### Enhancing non-invasive diagnostics through AI

AI has already demonstrated its potential in improving noninvasive diagnostic tools in cardiology, particularly in imaging techniques such as echocardiography, CT, and MRI. AI algorithms can detect subtle abnormalities in cardiac imaging that may be missed by human experts, leading to more accurate and early diagnoses. Future research should prioritize the development of AI models capable of enhancing the diagnostic accuracy of these imaging techniques across various patient populations.

One area for future investigation is the integration of AI with portable diagnostic tools, enabling the real-time interpretation of imaging results in remote or underserved areas. This would be particularly useful in low-resource settings where access to specialized cardiovascular care is limited. Moreover, AI systems could be further developed to provide instant feedback during procedures such as cardiac catheterization, assisting cardiologists in making informed decisions in real time. Additionally, AI could play a critical role in improving the accuracy of noninvasive testing for coronary artery disease, such as FFR-CT. Continued research on the use of AI in refining these techniques could reduce the need for invasive diagnostic procedures, improve patient safety, and reduce healthcare costs^[^35^]^.

#### AI-enhanced wearable devices for continuous monitoring

Wearable devices such as smartwatches and implantable sensors are increasingly used for continuous heart monitoring, providing real-time data on parameters such as heart rate, rhythm, and oxygen levels. AI’s integration of AI with these devices has enabled early detection of cardiac arrhythmias, atrial fibrillation, and other abnormalities that might otherwise go unnoticed. As AI technology advances, research should focus on improving the accuracy of these devices and expanding their capabilities to monitor additional cardiovascular parameters such as blood pressure and cholesterol levels.

Future studies should explore how AI can leverage data from multiple wearable devices to create a more holistic picture of a patient’s cardiovascular health. By aggregating data from various sources, AI can provide a more comprehensive assessment of a patient’s risk of cardiovascular events and suggest personalized interventions. Additionally, research is needed to assess the long-term effects of continuous monitoring on patient outcomes, including whether AI-enhanced wearable devices lead to better management of chronic cardiovascular conditions, such as hypertension and heart failure^[^36^]^.

#### Addressing ethical and privacy concerns

As AI becomes more integrated into cardiology, addressing ethical concerns related to data privacy, transparency, and bias becomes critical. Future research should focus on developing ethical frameworks for the use of AI in cardiovascular care, particularly for the collection and analysis of patient data. Ensuring that AI systems comply with data protection regulations, such as the General Data Protection Regulation (GDPR) and the Health Insurance Portability and Accountability Act (HIPAA), is essential in maintaining patient trust. Moreover, there is a need for more research on mitigating biases in AI algorithms, especially those trained on datasets that may not adequately represent diverse populations. By developing bias detection tools and improving dataset diversity, AI systems can become more equitable and reliable across different demographic groups.

Research is also needed to explore how AI can maintain transparency in its decision-making process. As AI algorithms become increasingly complex, it can be challenging for clinicians to understand how certain recommendations or diagnoses are generated. Developing explainable AI models that allow clinicians to trace the rationale behind AI-driven decisions is crucial for ensuring accountability and maintaining clinician trust in these technologies. AI has been poised to transform the field of cardiology by improving diagnostic accuracy, enhancing predictive models, and enabling continuous monitoring through wearable devices. However, to fully realize the potential of AI in cardiovascular care, significant research is required to address the current gaps. This includes advancing predictive analytics, refining noninvasive diagnostic tools, improving wearable device technology, and addressing the ethical challenges posed by AI integration. By focusing on these areas, AI can be harnessed to provide personalized, accurate, and effective cardiovascular care while maintaining the highest ethical standards^[^37^]^.

### Gaps

The integration of AI into cardiology has introduced groundbreaking innovations that hold promise for improving patient care, diagnostics, and management. Despite these advances, significant research and clinical gaps remain to be addressed to ensure the effective and equitable application of AI in cardiology. These gaps span issues of bias, need for better impact assessments, and development of techniques to enhance the interpretability and explainability of AI systems. Addressing these gaps is essential for building trust in AI-powered healthcare and for ensuring that these technologies are applied in ways that benefit all patients.

#### Understanding the sources of bias in AI systems

A major challenge in the use of AI in cardiology is the risk of bias within AI algorithms, which can lead to inequitable outcomes in different patient populations. AI models rely on large datasets to make predictions; however, these datasets may not adequately represent the full diversity of patient demographics. Underrepresentation of certain populations, such as ethnic minorities, women, and individuals from lower socioeconomic backgrounds, can skew the results of AI-powered diagnostics and recommendations. The source of this bias often lies in the data used to train the AI systems. Many datasets are derived from clinical trials and studies that do not reflect real-world populations, particularly in terms of diversity. For instance, cardiovascular diseases are known to disproportionately affect certain demographic groups, yet these groups are often underrepresented in the training datasets. As a result, AI models may not perform well for these populations, perpetuating health disparities. Further research is needed to identify these sources of bias and address them through the inclusion of more comprehensive and diverse datasets that better represent the global population.

#### Impact assessment of bias on clinical outcomes

While there is a growing awareness of the existence of bias in AI systems, its impact on clinical outcomes in cardiology is not fully understood. A key gap in the current research is the lack of thorough investigations into how biased AI systems affect healthcare delivery across different demographic areas. This gap is particularly relevant in cardiology, where accurate diagnosis and timely interventions can be a matter of life and death. For example, an AI model that is less accurate in predicting cardiovascular events in underrepresented populations could result in delayed diagnoses, inappropriate treatments, or unnecessary interventions. These missteps could lead to worsened outcomes for these patients, exacerbating the existing healthcare inequalities. Rigorous studies are required to assess the real-world impact of AI bias on clinical outcomes, particularly in underserved and minority populations. Such assessments would provide critical insights into how AI technologies can be improved to deliver equitable care to all patients regardless of their background.

#### Prioritizing explainability and interpretability of AI systems

Another significant research gap in AI-driven cardiology is the lack of transparency in how these systems generate output. Many AI models, particularly those based on deep learning, operate as “black boxes, “where the decision-making process is not easily understandable, even to clinicians. This lack of interpretability raises concerns about the trustworthiness and safety of AI systems, especially when they are used in high-stake medical decision-making. Clinicians and patients may be hesitant to rely on AI recommendations if they cannot understand how the system arrived at a particular conclusion. This lack of transparency not only undermines trust, but also poses challenges for clinical accountability. If an AI system makes a mistake, it is difficult to trace the origin of the error without a clear understanding of the inner workings of the model. Thus, there is an urgent need for research that prioritizes the development of techniques to enhance the interpretability and explainability of AI systems. Efforts to make AI models more transparent would improve clinician confidence in AI-driven diagnostics and treatment recommendations, ultimately leading to a better integration of AI into routine cardiology practice. Moreover, studies that investigate how explainability impacts clinician and patient trust in AI systems would provide valuable insights into how these technologies can be optimized for real-world applications.

#### Exploring techniques for improved explainability

Enhancing the interpretability of AI models is crucial to gaining clinician and patient trust. Techniques such as attention maps, model simplifications, and hybrid AI models that combine machine learning with rule-based systems can help make AI systems more understandable. Research is needed to explore how these techniques can be integrated into current AI models without sacrificing their accuracy. Furthermore, future studies should assess how improving the transparency of AI systems affects their acceptance in clinical settings. Explainability is particularly important in cardiology, where life-saving decisions are often made quickly, based on recommendations generated by AI. Trust in AI systems can only be built when both clinicians and patients are confident in the reliability of the technology.

Although AI holds immense potential to transform cardiology, several research and clinical gaps remain that must be addressed for its successful implementation. Understanding the sources of bias in AI systems and evaluating the impact of this bias on clinical outcomes are critical for ensuring equitable healthcare delivery. Moreover, prioritizing explainability and transparency in AI models is essential for building trust in these technologies and seamlessly integrating them into clinical practice. By addressing these gaps, AI can be harnessed to improve cardiovascular care in a way that is both effective and ethical.

## Conclusion

In summary, using machine learning AI algorithms can decrease the overreliance and burden on cardiologists by facilitating ECG evaluation. It is vital to devise cost-effective solutions and funding models to facilitate the integration of AI into cardiology. Comprehensive provider education and emphasizing AI’s role of AI as a tool to augment and not replace their expertise can address these concerns. Building regulatory protocols that can balance AI development with patient protection is paramount for the effective use of AI. Ensuring that AI is accurate and dependable involves validation and continuous performance tracking. Adherence to data protection laws and the establishment of robust security measures in place, such as encryption and access restrictions, are essential. Incorporating fairness includes generating newer generations of algorithms with built-in auto-detection of bias and auto-correction mechanisms and ensuring diverse representations in training modules. Privacy in healthcare organizations is essential for preserving patient trust and ethical data-handling practices. All of these factors can help overcome the challenges in AI-driven cardiology. AI can be used by cardiologists to improve the accuracy of interpretations and enhance their efficiency and can hence assist in providing better care to patients.

## Data Availability

Not applicable.
